# Exploring the Impact of BK_Ca_ Channel Function in Cellular Membranes on Cardiac Electrical Activity

**DOI:** 10.3390/ijms25031537

**Published:** 2024-01-26

**Authors:** Yin-Chia Chen, Chia-Lung Shih, Chao-Liang Wu, Yi-Hsien Fang, Edmund Cheung So, Sheng-Nan Wu

**Affiliations:** 1 Division of Cardiovascular Surgery, Department of Surgery, Ditmanson Medical Foundation Chia-Yi Christian Hospital, Chiayi City 60002, Taiwan; 07823@cych.org.tw; 2 Clinical Research Center, Ditmanson Medical Foundation Chia-Yi Christian Hospital, Chiayi City 60056, Taiwan; 15063@cych.org.tw; 3 Ditmanson Medical Foundation Chia-Yi Christian Hospital, Chiayi City 60002, Taiwan; wumolbio@mail.ncku.edu.tw; 4 Institute of Clinical Medicine, National Cheng Kung University Hospital, College of Medicine, National Cheng Kung University, Tainan 70403, Taiwan; eddiefang0023@gmail.com; 5 Department of Anesthesia, An Nan Hospital, China Medical University, Tainan 70965, Taiwan; edmundsotw@gmail.com; 6 Department of Research and Education, An Nan Hospital, China Medical University, Tainan 70965, Taiwan; 7 School of Medicine, College of Medicine, National Sun Yat-sen University, Kaohsiung 80421, Taiwan

**Keywords:** large-conductance Ca^2+^-activated K^+^ channel, Ca^2+^-activated K^+^ current, fibroblast, membrane potential, cardiac action potential

## Abstract

This review paper delves into the current body of evidence, offering a thorough analysis of the impact of large-conductance Ca^2+^-activated K^+^ (BK_Ca_ or BK) channels on the electrical dynamics of the heart. Alterations in the activity of BK_Ca_ channels, responsible for the generation of the overall magnitude of Ca^2+^-activated K^+^ current at the whole-cell level, occur through allosteric mechanisms. The collaborative interplay between membrane depolarization and heightened intracellular Ca^2+^ ion concentrations collectively contribute to the activation of BK_Ca_ channels. Although fully developed mammalian cardiac cells do not exhibit functional expression of these ion channels, evidence suggests their presence in cardiac fibroblasts that surround and potentially establish close connections with neighboring cardiac cells. When cardiac cells form close associations with fibroblasts, the high single-ion conductance of these channels, approximately ranging from 150 to 250 pS, can result in the random depolarization of the adjacent cardiac cell membranes. While cardiac fibroblasts are typically electrically non-excitable, their prevalence within heart tissue increases, particularly in the context of aging myocardial infarction or atrial fibrillation. This augmented presence of BK_Ca_ channels’ conductance holds the potential to amplify the excitability of cardiac cell membranes through effective electrical coupling between fibroblasts and cardiomyocytes. In this scenario, this heightened excitability may contribute to the onset of cardiac arrhythmias. Moreover, it is worth noting that the substances influencing the activity of these BK_Ca_ channels might influence cardiac electrical activity as well. Taken together, the BK_Ca_ channel activity residing in cardiac fibroblasts may contribute to cardiac electrical function occurring in vivo.

## 1. Physiological Implications of Large-Conductance Ca^2+^-Activated K^+^ (BK_Ca_) Channels

The large-conductance Ca^2+^-activated K^+^, commonly known as BK_Ca_ or BK channels—alternatively referred to as big Ca^2+^-activated K^+^ channels—are also recognized by names such as KCa1.1, KCNMA1, or *Slo*1 channels. These channels consist of four pore-forming α subunits, each containing seven transmembrane segments (S0–S6), contributing to their distinctive structure. Unlike voltage-gated K^+^ (K_V_) channels, the S0 segment is positioned at the N-terminus of each of the four pore-forming α subunits, while the S1–S6 segments collectively form the transmembrane region of the BK_Ca_ channel. 

**Figure 1:**
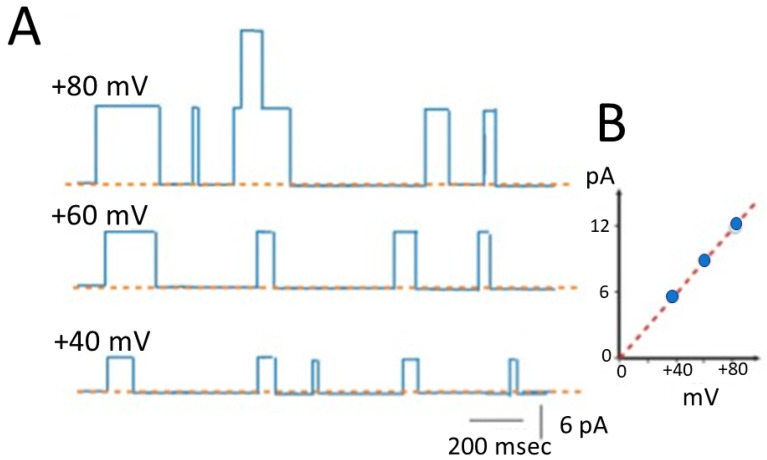
Biophysical characteristics of large-conductance Ca^2+^-activated K^+^ (BK_Ca_) channels. The examined cells were bathed in a symmetrical K^+^ solution (145 mM K^+^) with a reversal potential of around 0 mV. (**A**) Idealized traces of BK_Ca_ channels in a cardiac fibroblast. These channels display dynamic behavior as they transition between open and closed states, and these state transitions occur randomly. The dashed orange line in each current trace serves as the reference point for zero current, signifying the channel’s closed state. The upper deflection of each current trace indicates events of random opening of the ion channel. The voltage values in the upper left corner of each current trace represent the level of the holding potential applied. Of note, as the voltage becomes more positive, there is an increase in both the amplitude and the open-state probability of ion flow through the BK_Ca_ channel. The traces in Figure 1A and Figure in Section 1.2 were idealized using the QUB package (https://qub.mandelics.com/), accessed on 5 January 2024. (**B**) Current versus voltage (*I*–*V*) relationship of single BK_Ca_ channels with a reversal potential of 0 mV. The experiments were conducted in human cardiac fibroblasts, cells were bathed in a symmetrical K^+^ solution, and the recording pipette was filled with a K^+^-enriched solution. The reversal potential of K^+^ ions refers to the membrane potential at which there is no net flow of K^+^ ions across the cell membrane. Filled blue circles represent the measured amplitudes of BK_Ca_ channels. The dashed red line indicates a linear relationship, specifically the slope of the line, and thus provides an approximate single-channel conductance value of approximately 150 pS [13].

BK_Ca_ channels are members of the K_V_ channel family, distinguished by their unique mode of activation. Activation of these channels occurs through an allosteric mechanism triggered by alterations in intracellular Ca^2+^ levels, variations in membrane potentials, or a combination of both factors [1,2,3,4]. Upon random activation, BK_Ca_ channels facilitate the flow of substantial amounts of ions, specifically favoring highly selective K^+^ ions, through the cell membrane. Additionally, owing to their substantial conductance or permeability to K^+^ ions, with single-channel conductance typically ranging from approximately 150 to 250 pS, BK_Ca_ channels are classified as maxi- or large-K^+^ channels, as illustrated in Figure 1. The single-channel conductance of BK_Ca_ channels refers to the measure of K^+^ ion flow through a single open channel of this type. 

This family of voltage-gated K^+^ (K_V_) channels is functionally expressed in a broad spectrum of both excitable and non-excitable cell types. Alterations in the activity of BK_Ca_ channels can result in fluctuations in the magnitude of Ca^2+^-activated K^+^ currents (*I*_K(Ca)_) within the cell, thereby influencing the cell’s membrane potential. These channels demonstrate varying levels of abundance across diverse tissues in the body, including but not limited to smooth muscle tissues, neurons, endothelial cells, adrenal chromaffin cells, epithelial tissues, and pancreatic β cells [1,2,3,4,5]. They are ubiquitously expressed and play diverse roles in the regulation of cellular functions. Their activity has been shown to be involved in numerous physiological or pathological processes. These include membrane excitability, Ca^2+^ signaling, hormone or neurotransmitter release, stimulus-secretion coupling, muscle relaxation, and motor coordination [1,3,4,5,6,7,8,9,10]. However, the specific roles and levels of abundance can vary between tissues and under different physiological or pathological conditions. Numerous natural and synthetic molecules have been also shown to play crucial roles in regulating BK_Ca_ channel activity [11,12]. It has been established that alterations in BK_Ca_ channel activity can significantly impact cardiac function [13,14,15]. Furthermore, this review paper presents substantial evidence showing the presence of functional BK_Ca_ channels on the cell membranes within cardiac tissue. Importantly, the modulation of these ion channels can result in changes in the electrical activity of heart tissue.

### 1.1. The Role of BK_Ca_ Channel Activity Residing in Cardiac Fibroblasts

Fibroblasts, residing in connective tissue, are pivotal for synthesizing the extracellular matrix and collagen, thereby offering structural support to tissues, and playing a critical role in the process of wound healing. Specifically, cardiac fibroblasts, located within the heart tissue, are responsible for both the synthesis and maintenance of the extracellular matrix, which is indispensable for providing essential structural support to the heart. This matrix serves as a foundational framework for cardiomyocytes, the muscle cells responsible for the heart’s contraction. 

Unlike cardiac muscle cells (cardiomyocytes), which are responsible for rhythmic and coordinated contraction, cardiac fibroblasts, classified as connective tissue cells, are indispensable for upholding the structural integrity of the heart. This is achieved through the synthesis of collagen and other proteins located within the myocardial tissue of the heart. Functional integrity of the heart tissue pertains to the overall health, stability, and proper operation of the various components comprising the physical structure of the heart. The well-developed heart is a complex organ composed of different types of cells, including cardiomyocytes, fibroblasts, blood vessels, and extracellular matrix.

BK_Ca_ channels undergo substantial activation in response to either depolarization of the cell membrane or an increase in intracellular Ca^2+^ levels. Furthermore, the whole-cell *I*_K(Ca)_, where BK_Ca_ channel activity is relevant, displays a pronounced outward rectifying characteristic [3,13,16]. In the context of K_V_ channels, outward rectification means that the channel primarily allows ions to move out of the cell in response to changes in membrane potential. In other words, the BK_Ca_-channel’s conductance is higher for K^+^ ions moving in the outward direction (from inside the cell to the extracellular space) compared to K^+^ ions moving in the inward direction (from outside the cell to the intracellular space). This characteristic is significant in functions such as membrane hyperpolarization or repolarization, shaping action potentials, and the modulation of smooth muscle contraction [2,3,17]. 

However, owing to the outward rectifying property of BK_Ca_ channels, their functionality is relatively minimal when the cell membrane is at its resting potential. Consequently, in non-excitable cells such as fibroblasts, where changes in the cell membrane potential are minimal, the impact of BK_Ca_ channels on these cells seems to be constrained. In other words, if cardiac fibroblasts do not generate a heart-like action potential, their resting membrane potential is supposed to be roughly between −20 and −40 mV. In this case, BK_Ca_ channel activity is very weak. However, through effective electrical coupling, fibroblasts transition to having action potentials, causing the membrane potential to depolarize and consequently activate more BK_Ca_ channels. As a result, the magnitude of the whole-cell Ca^2+^-activated K^+^ current in cardiac fibroblasts increases significantly.

The action potential in the ventricles of mammalian hearts, excluding rodents, typically manifests as a square- or dome-shaped waveform, characterized by the presence of a plateau potential. The ventricular action potential refers to the sequence of electrical events that occur in the cardiac ventricular cells during each heartbeat. This process involves a series of phases, each characterized by specific changes in membrane potential. The interplay of specific ionic currents during the ventricular action potential ensures the proper coordination of electrical events leading to contraction and relaxation of the ventricular tissues.

It is worth noting, however, that a mitochondrial BK_Ca_ channel has been previously identified in dermal fibroblasts and heart cells [15,18,19]. These channels are located within the inner mitochondrial membrane, where they contribute to the regulation of mitochondrial function and cellular bioenergetics [10,20].

BKCa channel activity has been observed to contribute to membrane hyperpolarization in vascular endothelial cells, as part of endothelium-derived processes [10,20]. It is important to note that, unlike vascular smooth muscle cells, where the BK_Ca_ channels employ a negative feedback mechanism to regulate the excessive increase of intracellular Ca^2+^ ions, in vascular endothelial cells, the functioning of BK_Ca_ channels in vascular endothelial cells relies on positive feedback mechanisms to control the intracellular elevation of Ca^2+^ ions. Much like vascular or cardiac fibroblasts, a significant portion of vascular endothelial cells do not exhibit electrical excitability. Consequently, the control of intracellular Ca^2+^ within these cells relies on an electrochemical driving force, with a particular emphasis on the flux of Ca^2+^ ions. In simpler terms, the voltage gradient and concentration gradient of Ca^2+^ both align inward in these cells. The main reason for this is that the concentration of Ca^2+^ outside the cell is a thousand times greater than inside the cell, and Ca^2+^ itself carries a double positive charge. The electrochemical driving force refers to the combined influence of both the electrical gradient and the chemical (concentration) gradient acting on ions across a cell membrane. Resting intracellular Ca^2+^ concentrations in fibroblasts or endothelial cells are typically maintained at low levels within the cytoplasm, usually around 100 nM. The concentration of extracellular Ca^2+^ usually ranges from approximately 1.1 to 1.3 mM in the blood plasma. However, in response to stimulation, such as exposure to diverse signaling molecules or mechanical forces, a swift and transient elevation in intracellular Ca^2+^ levels can occur. Furthermore, the activation of BK_Ca_ channels is modulated by localized microdomains beneath the surface membrane. The precise intracellular concentration of Ca^2+^ concentration necessary for BK_Ca_ channel activation may vary contingent on the specific tissue or cell type.

However, in electrically excitable cells, such as vascular smooth muscle cells, a negative feedback mechanism operates, revealing a complex interplay during membrane depolarization. When membrane depolarization induces voltage-gated Ca^2+^ currents—such as T- or L-type Ca^2+^ currents—across the cell membrane, extracellular Ca^2+^ ions can readily ingress the cell. Concurrently, membrane depolarization activates voltage-gated Na^+^ current, further promoting cell depolarization. This dual effect, characterized by both membrane depolarization and elevated cytosolic Ca^2+^ concentrations, triggers the activation of BK_Ca_ channels. Consequently, these channels lead to membrane hyperpolarization by facilitating the efflux of K^+^ ions out of the cell. This hyperpolarization, in turn, results in the inactivation of voltage-gated Na^+^ and Ca^2+^ currents [2,3,21]. The activation of BK_Ca_ channels thus contributes to a retardation in the elevation of intracellular Ca^2+^, ensuring meticulous regulatory control.

The hERG or Kv7.1 channels indeed play a crucial role in the repolarization of the cardiac action potential. However, in mature cardiomyocytes, BK_Ca_ channel activity is absent. Consequently, how the activity of BK_Ca_ channels compares with other K^+^ channels in influencing the repolarization of mature cardiomyocytes remains unknown. It may be necessary to investigate whether effective electrical coupling, both qualitatively and quantitatively, can occur between these cardiac cells and the surrounding fibroblasts to gain insights into this aspect.

**Figure 2:**
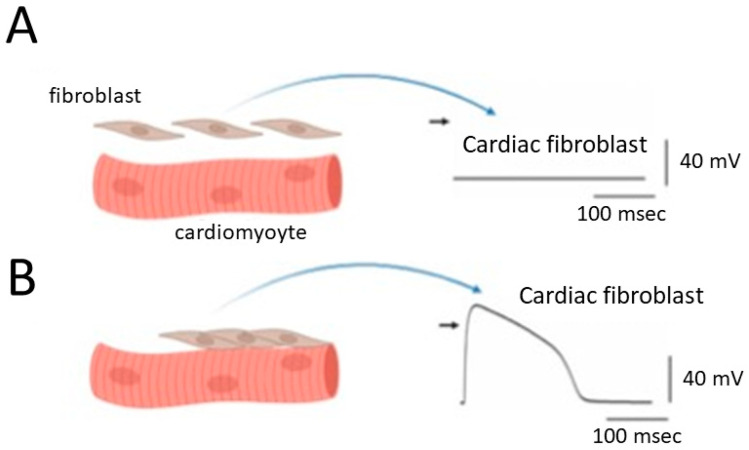
Alterations in the membrane potential of cardiac fibroblasts when they are structurally and electrically coupled to neighboring cardiomyocytes. Panel (**A**) represents this scenario in the absence of coupling, while panel (**B**) shows the situation in the presence of effective structural and electrical coupling. The graph depicts cardiac myocytes on the left side, showcasing evident striations. Importantly, the diagram highlights cardiac cells characterized by distinct striations, serving as an indicator of ventricular cells within the heart. Additionally, a comparable coupling phenomenon is observed in cardiac atrial cells when a sufficient number of large fibroblasts are present in the atrium. The black short arrow on the right side of (**A**,**B**) indicates the zero potential level. The vertical and horizontal vars in the bottom right corner of each potential trace represent the calibration marks [13].

A noteworthy finding highlights the existence of BK_Ca_ channels in cardiac fibroblasts [13,14,22]. These channels are thought to play a role in facilitating potential electrical coupling between myocyte and fibroblast. Electrical coupling denotes the direct electrical link between neighboring cells, and computational modeling studies in silico lend support to this phenomenon [13,23] (Figure 2). This coupling is often used in the context of neurons or certain types of muscle cells where rapid and synchronized communication is essential. When cardiac fibroblasts are numerous and establish robust connections with neighboring cardiac cells through gap junctions or intercalated discs, functional electrical coupling can be ensured. Gap junctions, protein channels spanning the cell membranes of adjacent cardiomyocytes, are thought to allow for the direct exchange of ions (such as Na^+^, K^+^, and Ca^2+^ ions) and small molecules between neighboring cells [24]. Connexins are a family of proteins that play a critical role in the formation of gap junctions, specialized intercellular channels that allow direct communication between adjacent cells. These gap junctions enable the exchange of ions, small molecules, and signaling molecules, contributing to the coordination of physiological functions in tissues. 

Moreover, the intercalated discs in heart tissues are specialized structures found in the heart tissue that play a crucial role in facilitating communication and coordination between adjacent cardiac muscle cells of the heart. This can allow them to work together as a functional unit during the contraction and relaxation of the heart. These discs play a crucial role in maintaining the integrity of the cardiac tissue, ensuring the synchronization of both electrical signals and mechanical forces. This synchronization is essential for the efficient and coordinated contraction of the heart. In other words, cardiomyocytes and fibroblasts may form a functional syncytium, allowing for coordinated contraction and efficient pumping of blood. The functional syncytium pertains to the unified and synchronized contraction of cardiac muscle cells in the heart tissue. The ability of these cells to form a functional syncytium is facilitated by their electrical connectivity through gap junctions.

In this context, cardiac fibroblasts may display a “dome-like configuration” in their action potentials, referencing the unique shape observed in specific cardiac cell types, particularly ventricular cardiomyocytes [13,25,26,27] (Figure 2). 

Consequently, the depolarization of fibroblasts becomes more pronounced, emphasizing the crucial role of BK_Ca_ channels in cardiac fibroblasts due to their outwardly rectifying property. This prompts an intriguing hypothesis that cardiac fibroblasts may engage in significant interactions with cardiomyocytes, potentially making a significant contribution to the heart’s overall structural and functional integrity (Figure 2). This hypothesis becomes especially pertinent when contemplating the potential augmentation of their numbers or sizes within cardiac tissues, particularly in conditions such as atrial fibrillation or preceding myocardial infarction [22,28,29,30,31,32]. Atrial fibrillation is a common and often chronic heart rhythm disorder that affects the upper chambers of the heart, known as the atria. In atrial fibrillation, the normal coordinated electrical impulses that regulate the heart’s rhythm become chaotic and irregular. Consequently, instead of the atria contracting efficiently to move blood into the ventricles, they quiver or fibrillate. The preceding or old myocardial infarction, commonly referred to as an “old heart attack”, is a term used to describe a previous heart attack or myocardial infarction that occurred in the past, and the affected tissue has undergone certain changes over time.

Cardiac fibroblasts play a crucial role in the process of cardiac remodeling that ensues after an old myocardial infarction. Following a myocardial infarction, a section of the heart muscle experiences oxygen deprivation, leading to the demise of cardiomyocytes. Subsequently, the body initiates a sequence of reparative and adaptive processes involving the activation of various cells, including cardiac fibroblasts [25,28,29]. Furthermore, cardiac remodeling, which encompasses both structural and functional alterations in response to diverse stimuli or stressors, relies on the functional capacity of cardiac fibroblasts. This process involves alterations in the size, shape, and function of the heart and its components. These adaptive changes are mechanisms aimed at preserving cardiac output and function in the face of altered conditions, such as increased workload, injury, or pathological circumstances. However, if the remodeling process becomes excessive or prolonged, namely pathological remodeling, it can result in functional impairment, potentially leading to conditions like heart failure. This compromise in the heart’s pumping ability may increase the risk of arrhythmias.

### 1.2. Influence of BK_Ca_ Channel Activity in Cardiac Fibroblasts on Membrane Potential of Heart Cells 

Mammalian heart cells display a diverse cellular composition, placing primary emphasis on cardiomyocytes, cardiac fibroblasts, and endothelial cells. Notably, among the non-myocytes, cardiac fibroblasts are not electrically excitable and may constitute a substantial proportion, particularly in cases of atrial fibrillation or old myocardial infarction. These cardiac fibroblasts have been suggested to play crucial roles in shaping both the structure and functionality of the myocardium [26,29]. It is believed that these fibroblasts contribute to various aspects of cardiac performance, encompassing structural, biochemical, mechanical, and even electrical dimensions [25,26,29].

Although fully mature mammalian heart cells display electrical excitability, they do not possess functional activation of BK_Ca_ channels, despite the consistent functional expression of voltage-gated Na^+^ and Ca^2+^ currents in cardiac cells. Conversely, undifferentiated cardiomyocytes derived from embryonic stem cells have been observed to express the activity of these channels which is sensitive to be increased by membrane depolarization and/or elevated cytosolic Ca^+^ concentration [33,34]. The function of these channels in undifferentiated cardiomyocytes is also responsive to inhibition by paxilline, a natural product and a class of indole alkaloids known to effectively suppress the activity BK_Ca_ channels, but not by apamin, a blocker of small-conductance Ca^2+^-activated K^+^ channels. A contributing factor to this difference is the relatively prolonged duration of action potentials with a plateau potential in mature heart cells, which is attributed to the absence of functionally active BK_Ca_ channels [1,2]. Likewise, cardiomyocytes differentiated in vivo from amniotic fluid-derived stem cells showed no significant activity of BK_Ca_ cells [33]. Alternatively, earlier studies have suggested the possible presence of KCa1.1 (or KCNMA1) in human sinus node function [35,36]. 

**Figure 3:**
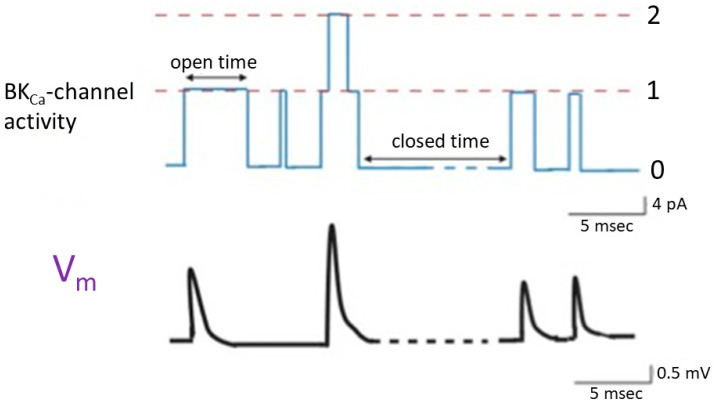
Changes in membrane potential (**lower** panel) triggered by the random opening of idealized BK_Ca_ channels (**upper** panel) inherently in cardiac fibroblasts. The simultaneous cell-attached potential and current recordings were made in this study, and cells were immersed in normal Tyrode’s solution which contained 5.4 mM K^+^, and 1.8 mM Ca^2+^. Tyrode’s solution is a physiological salt solution used in biological and medical research to mimic the extracellular fluid environment in which cells are studied. The dashed orange line in the upper panel represents each level of BK_Ca_-channel opening, while the numerical values on the right indicate the number of channel openings. The double arrow in the channel activity indicates variable duration in the opening or closed state. The upper panel represents the activity of BK_Ca_ channels. The lower panel shows the changes in membrane potential induced by BK_Ca_-channel activity. Upward deflection in current (blue color) and potential (black color) traces represents the occurrence of channel opening and membrane depolarization, respectively. In the bottom right corner of both the upper and lower panels, there are calibration marks. Of note, the occurrence of a transient depolarization is not triggered by the second channel opening event, as this event exhibits a brief duration of channel openness, which is less than 1 msec. This figure was modified from a paper [33].

Prior studies have shown that in cell-attached current-clamp voltage recordings, the sporadic opening and closure of BK_Ca_ channels were robustly observed in cardiac fibroblasts. The activity of these channels has the potential to induce random depolarizing waveforms via effective electrical coupling [33,37,38] (Figure 3). This phenomenon was also previously found in bovine chromaffin cells [39]. On the other hand, when heart cells are near cardiac fibroblasts, the activity of BK_Ca_ channels present on the surface of these fibroblasts can lead to varying degrees of membrane depolarization. When the cell membranes of these cardiac cells undergo random depolarization, coupled with the accumulation of temporal and spatial phenomena, it is likely to significantly increase the excitability of the cell membrane, even inducing the generation of action potentials. If the BK_Ca_ channels on cardiac fibroblasts are activated due to the treatment with the openers of the channel [11,14,16,20], it might even result in the facilitation of subtle membrane depolarization in neighboring heart cells. This effect is attributed to effective electrical coupling between cardiac fibroblasts and neighboring heart cells. 

Additionally, there are reports of paxilline, which belongs to the indole-diterpene class of mycotoxins and is recognized as a potent inhibitor of BK_Ca_ channels. Paxilline is a toxin produced by certain species of fungus, particularly by the mushroom *Paxillus involutus* [13,40], leading to a reduction in heart rate [35]. Therefore, exploring the potential impact of diverse BK_Ca_ channel modulators on the regulation of cardiac electrical activity is also a worthwhile pursuit. However, it is noteworthy that paxilline can inhibit sarco/endoplasmic reticulum Ca^2+^ ATPase (SERCA) [41], a crucial player in excitation-contraction coupling and cardiac muscle contraction. The precise contribution of paxilline to heart rate effects requires clarification, as it remains uncertain whether these effects are primarily attributed to the modulation of BK_Ca_ channels or the inhibition of SERCA activity. 

### 1.3. BK_Ca_-Channel Activity Residing in Vascular Smooth Muscle Cells

Alternatively, in electrically excitable cells like vascular smooth myocytes and pituitary GH_3_ lactotrophs, there is compelling evidence suggesting that the activity of BK_Ca_ channel activity can potentially interact with voltage-gated Ca^2+^ (Ca_V_) channels. This interaction leads to the formation of BK_Ca_-Ca_V_ complexes, facilitating rapid and localized Ca^2+^-activated K^+^ signaling [3,17,21,42]. The expression of auxiliary β-subunits was also reported to be lacking in BK_Ca_-channels within cardiac fibroblasts; however, these subunits (i.e., α + β subunits) are abundant in vascular muscle cells. 

Previous studies have also shown that BK_Ca_ channels in vascular smooth muscle cells exhibit diverse increases in activity when exposed to membrane stretching, even in conditions that simulate vasomotion through cyclic stretching [43]. Vasomotion involves rhythmic, spontaneous, and oscillatory changes in blood vessel diameter, particularly in small arterioles and venules. The frequency of vasomotion can vary among individuals and across different vascular beds, commonly ranging from 0.02 to 0.3 Hz. Consequently, it is a plausible hypothesis that BK_Ca_-channel activity could impact the interaction between fibroblasts and vascular myocytes or cardiomyocytes through cyclic or pulsatile changes in mechanical stress [22,44]. Mechanical stress or strain in blood vessels refers to the physical forces exerted on the vascular walls as a result of the pressure and flow of blood within the circulatory system.

On the other hand, while mature cardiac cells lack functional expression in BK_Ca_ channels [1,8,40,41,42,45,46], the majority of vascular smooth muscle cells typically display significant activity in BK_Ca_ channels. The heightened BK_Ca_ channel activity residing in vascular smooth muscle cells is recognized as contributing to the occurrence of whole-cell spontaneous transient oscillatory outward currents (STOCs) in these cells, even in the absence of external stimulation [47,48,49]. Spontaneous transient outward currents often occur in different types of smooth muscle cells, such as vascular smooth muscle cells, aligning with the occurrence of spontaneous transient oscillatory membrane hyperpolarization observed during the current-clamp configuration. These occurrences involve brief, self-limiting episodes of membrane hyperpolarization arising from the activation of K_V_ channels, especially BK_Ca_ channels. Therefore, a thorough investigation is thus warranted to determine whether the increased activity of BK_Ca_ channels in vascular smooth muscle cells, especially at the junctions of blood vessels and cardiac tissues such as veins and the atria, or large arteries and the ventricles, might have a potential role in generating ectopic foci and subsequently triggering cardiac arrhythmias in these specific locations [7,22,31,32,43,45,50]. Indeed, earlier reports have identified the source of specific atrial tachycardia, flutter, or fibrillation, with many originating at the interface between the pulmonary veins and the posterior left atrium [51,52]. Therefore, the BK_Ca_ channel activity in this region may influence the occurrence of arrhythmias.

## 2. Conclusions

BK_Ca_ channels are ion channels that respond to fluctuations in intracellular Ca^2+^ levels by facilitating the efflux of K^+^ ions. This process influences cell membrane potential and various cellular functions across different tissues and cell types. These channels are particularly important in the regulation of cellular excitability, neurotransmitter or hormone release, smooth muscle tone, and cardiovascular function [2,3,7,8]. This paper underscores the significant influence of BK_Ca_ channel activity across the membrane of cardiac fibroblasts on the electrical dynamics of cardiac cells, especially in situations where these fibroblasts are plentiful and intricately connected with a substantial proportion of neighboring cardiac cells. 

This paper emphasizes that BK_Ca_ channels are notably absent in mature cardiomyocytes but are present in fibroblasts. However, due to intercellular connections, the presence of BK_Ca_ channels in fibroblasts can impact the membrane potential and action potential duration of neighboring cardiomyocytes. However, the connection between BK_Ca_ channels, the action potential, and the onset of arrhythmia seems insufficiently elucidated. Verification is essential for confirming whether alterations in BK_Ca_ channels can indeed disturb the action potential and induce arrhythmia in both cellular and animal model settings.

The walls of blood vessels, encompassing both arteries and veins, are composed of three primary layers known as the intima, media, and adventitia. The innermost layer is the intima, housing vascular endothelial cells, while the middle layer is the media, which contains vascular smooth muscle cells. Vascular fibroblasts are located in the connective tissue between the intima and media layers. Vascular endothelial cells are electrically non-excitable, whereas vascular smooth muscle cells are electrically active. This leads to speculation about the potential pivotal role of vascular fibroblasts in maintaining the structural, functional, and electrical integrity of blood vessels. Further research is necessary to explore the potential correlation between BK_Ca_ channel activity in these fibroblasts and the separation of intima and media, corresponding to the dissection of blood vessels [53]. The dissection of blood vessels refers to a condition where there is a separation or tearing of the layers that make up the walls of blood vessels. This separation can occur between the intima and media layers or within any of the vascular layers. It is commonly associated with diseases such as aortic or coronary artery dissection [54,55].

Moreover, it is noteworthy that, unlike heart tissues [13,24], there is a limited degree of electrical coupling between endothelial cells [25] and smooth muscle cells. For example, the basal lamina is a specialized extracellular matrix primarily associated with the endothelial cells of the intima layer, and offers structural support to these cells, effectively separating them from the underlying connective tissue. Therefore, within blood vessels, the electrical coupling occurs with varying efficiency among vascular endothelial cells, fibroblasts, vascular smooth muscle cells, and even ganglion cells or neurons. This variability is primarily attributed to the unequal distribution of connective tissue.

Alternatively, prior research has suggested that cardiac fibroblasts possess the ability to evoke additional types of whole-cell K^+^ currents elicited during membrane depolarization, demonstrating an outward rectifying property [22,23,29,45,56,57]. Previous studies have also highlighted the role of apamin-sensitive Ca^2+^-activated K^+^ (K_Ca_) channels, specifically small-conductance K_Ca_ channels, in exerting a proarrhythmic effect reported in isolated canine left atrium [58,59]. The activity of the BK_Ca_ channel is thought to be impervious to apamin, with susceptibility limited to iberiotoxin or paxilline. However, it remains unexplored whether the magnitude of these K_V_ currents similarly influences the membrane potential of neighboring heart cells, emphasizing the imperative for further investigation in this context [60].

## Data Availability

Not applicable.
